# Flame retardancy effects between expandable graphite and halloysite nanotubes in silicone rubber foam†

**DOI:** 10.1039/d1ra01409a

**Published:** 2021-04-13

**Authors:** Qingtao Pang, Furu Kang, Jun Deng, Lei Lei, Jie Lu, Shuiyuan Shao

**Affiliations:** College of Safety Science and Engineering, Xi'an University of Science and Technology Xi'an 710054 P. R. China kangfuru@stu.xust.edu.cn; Shaanxi Key Laboratory of Prevention and Control of Coal Fire, Xi'an University of Science and Technology Xi'an 710054 P. R. China dengj518@xust.edu.cn; College of Materials Science and Engineering, Xi'an University of Science and Technology Xi'an 710054 P. R. China

## Abstract

The effect of expandable graphite (EG) and modified halloysite nanotubes (HNTs) on the flame retardant properties of silicone rubber foam (SiF) was studied in this paper. Modified HNTs were obtained by surface modification of the silane-coupling agent A-171. The flame retardancy of SiF was studied by limiting oxygen index (LOI), vertical combustion and cone calorimeter tests. The mechanical properties of SiF were analyzed by a universal mechanical testing machine. The LOI results showed that EG/HNTS@A-171 could enhance the LOI of SiF. The cone calorimeter test results showed that EG/HNTS@A-171 effectively reduced the peak heat release rate, the total heat release rate, the smoke production rate, the total smoke production rate, the CO production rate and the CO_2_ production rate and increased the carbon residue rate. TGA shows that main chain pyrolysis temperature of the SiF is delayed by 123 °C. The mechanical properties test results showed that EG/HNTS@A-171 improved the tensile strength of SiF. These results indicated that EG/HNTS@A-171 can significantly improve the flame retardant performance of SiF.

## Abstract 2

The effect of expandable graphite (EG) and modified halloysite nanotubes (HNTs) on the flame retardant properties of silicone rubber foam (SiF) was studied in this paper.

## Graphical abstract

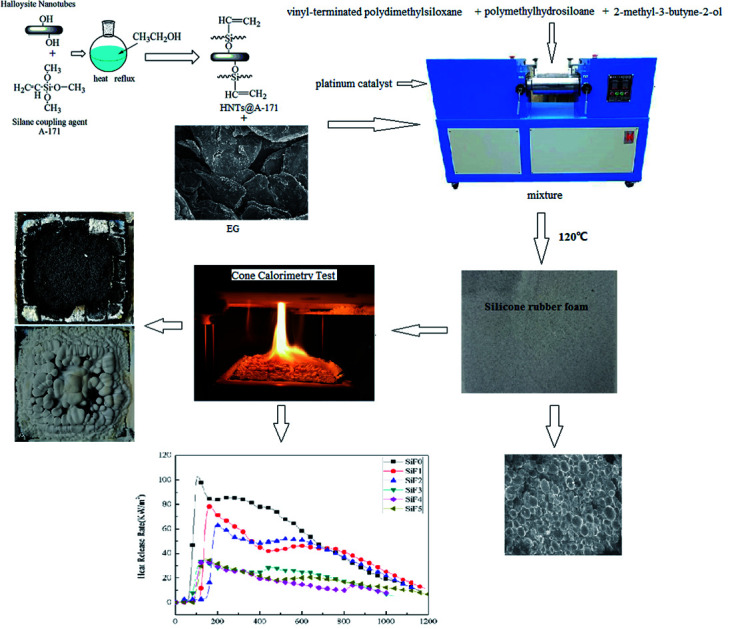

## 1. Introduction

SiF is a kind of porous material obtained from silicone rubber by physical and chemical foaming methods.^1^ It retains the unique electrical properties, chemical resistance, ultraviolet resistance and mechanical stability of silicone rubber^2–4^ and it has the advantages of low weight, heat insulation, flexibility and resilience of foam materials.^5,6^ Therefore, SiF and its composite materials have been widely used in the national defense and military industry and in aerospace, transportation, automobiles and other fields.^1,4,7^ In these fields, the flame retardancy requirement of materials is very high. Although silicone rubber is a semi-organic and semi-inorganic material, it has a low heat release rate, a low total heat release and a low ignition index.^8–10^ At the same time, the silicon rubber has excellent thermal stability and can be transformed into a continuous, oxidation resistant and insulating mesh silica fume that is covered on the surface after combustion,^11^ effectively preventing further ablation. However, once SiF has been ignited, it can continuously burn, especially the silicon rubber foam material, which is more vulnerable to combustion due to higher specific surface area, restricting its application in some fields.^12^ At present, the flame retardant properties of silicone rubber are mainly improved by adding hydroxide,^13^ clay material,^14^ phosphorous flame retardant,^15^ expansive flame retardant,^16^ GO^17,18^ and EG,^19^*etc.* However, there are few studies on the flame retardant properties of silicone rubber foam.

The flame retardancy of SiF is mainly improved through flame retardant compounds,^20^ microencapsulation^21^ and layer-by-layer self-assembly.^22^ Deng *et al.*^23^ studied the flame retardancy of SiF by compounding ultrafine aluminum hydroxide (ATH) with ultrafine calcium carbonate (CC). When 15 wt% ATH and 15 wt% CC were added, SiF was classified as UL-94 V0 grade, the LOI reached 35.2%, and the maximum smoke density decreased by 45%. Kang *et al.*^24^ applied superfine talc powder (SFT) hollow glass beads (HGBs) as flame retardants, and when 10 wt% SFT or 5 wt% HGBs was added, SiF was classified as The UL-94-V0. Kang *et al.*^21^ prepared a KH550-coated dimethyl methyl phosphonate (DMMP) microcapsule by means of microchannel UV curing using DMMP as the core and γ-methylacryloxy propyltrimethoxysilane (KH570) as the shell. When 15 wt% microcapsules were added, the SiF LOI reached 31.6%, and the material was classified as UL-94-V0. Deng *et al.*^22^ prepared CH nano-coated SiF by layered self-assembly with chitosan/ammonium polyphosphate (CH/APP) and chitosan/montmorillonite (CH/MMT). When the thickness was 7 layers, the LOI of the CH/APP-coated samples increased from 20.2% to 23.8%, the peak heat release rate decreased by 27.6%, and the total smoke product decreased by 42%. The total smoke product of the CH/MMT sample decreased by only 12%. All the above methods effectively improved the flame retardancy of SiF, but the large amount of addition and the poor flame retardant efficiency limited its wide application.

EG is a typical graphite intercalation compound. When heated, EG expands and generates a voluminous isolative layer, providing flame retardance for the polymeric matrix.^25–28^ In recent years, EG has been widely used in polyurethane,^29,30^ PP,^31,32^ PE,^33^ polystyrene,^34^ epoxy resin^35^ and other polymer flame retardant materials and has achieved satisfactory results. However, when EG is used alone in polymer flame retardation, the carbon layer containing EG is loose and porous, thus hindering perfect flame retardation performance. The vermicular structure layer formed by EG in the combustion process often lacks sufficient adhesion and is easily damaged by thermal convection and flame pressure in the combustion process.^36–38^ EG's flame-retardant can be improved by increasing the density of the carbon layer.

HNTs are a 1 : 1 aluminosilicate that can be found in nature, whose main property is its distinctive hollow tubular shape combined with its eco-compatibility, no-toxicity and low cost.^39,40^ Unlike kaolinite, halloite has a layer of adsorbed water between the layers, which can be released when heated. Besides, this, in combination with the flame retardant ability ascribed to noncombustible Si and Al elements, makes it feasible for HNT to be directly used for improving the fire safety of polymers. So, as nanomaterials, HNTs can not only improve the mechanical properties of polymers but also improve their flame retardant properties.^41–47^ As previously reported, the flame-retardant performance of HNT is mainly ascribed to their effect in the condensed phase. For instance, HNTs have been applied in the flame retardancy of epoxy resin,^48,49^ polybutanediol,^50^ cyanate resin,^51^ EPDM^52^ and other materials, and it is beneficial to improve the flame retardant properties of the polymer.

However, as far as we know, few research reports have focused on the mechanical properties and flame retardant properties of polymers with HNTs/EG. Vahabi *et al.*^53,54^ studied the flame retardant properties of EG and HNTs for epoxy resin composites respectively, and found that 3 wt% HNTs could improve the flame retardant properties and thermal stability of epoxy resin. Compared with EG sample, the flame retardant properties of HNTs were better. However, when 6 wt% or 9 wt% HNTs were added, the flame retardant properties were not as good as the same quality EG. Gillani *et al.*^55^ used polydimethylsiloxane (PDMS)/phenol BA epoxy resin as material to study the enhancement effect of halloite nanotubes (HNTs) on expanded graphite-based expanded fire-retardant coatings (IFRCs). By adding different weight percentage of HNTs and PDMS to the basic expansion components (ammonium polyphosphate/melamine/boric acid/expanded graphite, APP/MEL/BA/EG), the expansion coating formula was developed. The synergistic effect of HNTs and siloxane in the expansion reaction process enhances the polymer crosslinking in the binder system and improves the combustion performance.

The research on flame retardant properties of silicone rubber has a certain foundation. At the same time, EG and HNTs have been widely used in polymer materials as excellent flame retardants and some scholars have studied the flame retardant properties of EG and HNTs in epoxy resin system. However, they did not study the combination of the two, not to mention introduce them into the silicone foam material for systematic research. Because silicone rubber foam is based on Si–O–Si bond, which is different from epoxy resin, polyurethane and other polymer materials, it is of great significance to study the effect of EG and HNTS on the flame retardant properties of silicone rubber foam materials.

In this paper, based on EG/silicone rubber foam flame retardant system, EG has the defect of “popcorn effect”, proposed to use silane coupling agent A-171 to modify the surface of HNTs, to improve EG in the silicone rubber foam system “popcorn effect”. Silicone rubber foam was prepared by mechanical blending method after adding EG and HNTs@A-171 were mixed. The mechanical properties, flame retardant properties, residue morphology and thermal stability of SiF were studied.

## 2. Materials and methods

### 2.1. Materials

Vinyl-terminated polydimethylsiloxane, with a viscosity of approximately 150 000 mPa s and containing a vinyl group at a concentration of 0.0033 mol/100 g, the molecular weight and polydispersity are 2282 (MW) and 1.6, respectively, was purchased from Xi'an Daoson Chemical Technology Co., Ltd. (Shaanxi, China). A platinum complex with a platinum content of 3000 ppm was purchased from Shenzhen Osbang New Materials Co. (Guangdong, China); poly(methylhydrosiloxane) with a hydrogen content of 1.4 and an average Mn of 1700–3200 was purchased from Shanghai Macklin Biochemical Co., Ltd. (Shanghai, China) expandable graphite, with an average particle size of approximately 80 mesh, was purchased from Qingdao Yanhai Carbon Material Co., Ltd. (Shandong, China). Halloysite nanotubes were purchased from Shijiazhuang Huideli Mineral Products Co., Ltd. (Hebei, China). Vinyltrimethoxysilane (A-171) and 2-methyl-3-butyn-2-ol were purchased from Shanghai Macklin Biochemical Co., Ltd. (Shanghai, China). The expandable microsphere foaming agent F36D is a thermoplastic hollow polymer microsphere composed of a thermoplastic polymer shell and an enclosed liquid alkane gas, was purchased from Japan Matsumoto Oil and Fat Pharmaceutical Co., Ltd. The compositions of the mixtures are shown in Table 1.

**Table 1:** Formulations of flame-retardant SiF

Sample code	Vinyl-terminated polydimethylsiloxane/wt%	F36D/wt%	Poly(methylhydrosiloane)/wt%	Platinum complex/wt%	2-Methyl-3-butyn-2-ol/wt%	EG/wt%	HNTs@A-171/wt%
SiF0	100	10	2.0	0.2	0.01	0	0
SiF1	100	10	2.0	0.2	0.01	0	10
SiF2	100	10	2.0	0.2	0.01	10	0
SiF3	100	10	2.0	0.2	0.01	9	1
SiF4	100	10	2.0	0.2	0.01	7	3
SiF5	100	10	2.0	0.2	0.01	5	5

### 2.2. HNTs@A-171

**Fig. 1:**
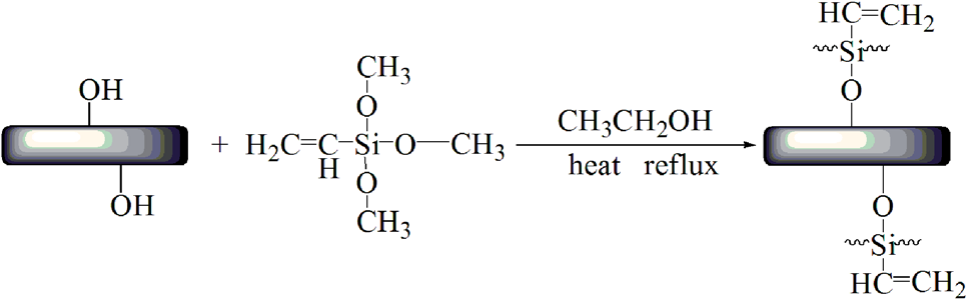
Preparation of HNTs@A-171.

First, 40 g HNTs and 400 g anhydrous ethanol were successively added to a 1000 mL three-mouth flask, and 10 g A-171 was added dropwise. The reaction was stirred at 70 °C and 400 rpm for 12 h. After filtration, the product was washed with ethanol 3 times, dried to a constant amount at 80 °C in a vacuum oven, and then grounded (Fig. 1).^56^

### 2.3. Preparation of samples

The preparation process of silicone rubber was as follows: firstly, vinyl trimethoxysilane, polymethylhydrosiloxane and 2-methyl-3-butyl-2-ol were weighed and added into the plastic cup. The pre-mixed sample was obtained after stirring for 3 min. Secondly, transfer the pre-mixed samples to the double-roll open mill (ZG-250, Dongguan Zhenggong Electromechanical Equipment Technology Co., Ltd), and mix the samples for 3 min at a 3 mm roll distance. Then, add silica, EG, HNTs and F36D and continue mixing for about 10 minutes. Further, the flame retardant silicone rubber semi-finished product was obtained by adding platinum catalyst and mixing for 5 min. Thirdly, adjust the roll distance more than 10 mm, at the same time, the flame retardant silicone rubber semi-finished product was quickly transferred from the opening mill to the constant volume grinding tool (the mould volume was 300.0 mm × 300.0 mm × 12.0 mm), and kept in the oven (model: DHG-9010, Suzhou Guangjun Electronic Technology Co., Ltd.) at 120 °C for 30 min, finally obtain the flame retardant silicone rubber sample.

In this experiment, poly(methylhydrosiloane) was used as a cross-linking agent, 2-methyl-3-butyn-2-ol was used as an inhibitor, platinum was used as a catalyst, F36D was used foaming agent, and EG and HNTs were used as flame retardants.

### 2.4. Characterization

The mechanical properties were determined on an electronic tensile testing machine (XZW5000N, Jiangsu Xinzhenwei Testing Machinery Co., Ltd., China) with a constant speed of 100 mm min^−1^ at room temperature according to GB/T 528-2009. Six samples were used in the mechanical properties test. And the mechanical properties value of each sample was the average value from five tests. The test sample is dumbbell type, and the sample test size was 25.0 mm × 4.0 mm × 3.0 mm.

The LOI was determined on a JF-3 oxygen index tester (Nanjing JiangNing Analytical Instrument Co., Ltd.) according to ISO4589. The sample size was 150.0 mm × 10.0 mm × 10.0 mm. Six samples were used in the LOI test. And the LOI value of each sample was the average value from five tests.

UL-94 was measured by a CZF-3 horizontal and vertical tester (Nanjing JiangNing Analytical Instrument Co., Ltd.) according to ASTMD3801-2010, and the sample size was 127.0 × 12.7 mm × 3.0 mm. Six samples were used in the UL-94 test.

Cone calorimetry was carried out using a cone calorimeter (Motis Fire Technology (China) Co., Ltd., Kunshan, China) under a heat flux of 35.0 kW m^−2^ by the ISO 5660 standard. All samples were 100.0 mm × 100.0 mm × 6.0 mm. Six samples were used in the cone calorimetry test. And the cone calorimetry value of each sample was the average value from three tests.

FT-IR test: infrared spectra of HNTS and HNTs@A-171 were determined by Bruker Vector 33 Fourier infrared spectrometer. The test wavelength range is 4000 cm^−1^ to 400 cm^−1^.

TGA was performed using a Synchronous Thermal Analyzer (TGA/DSC), (METTLER TOLEDO Instruments, Switzerland). At a heating rate of 20.0 °C min^−1^, 8.0–10.0 mg of the sample was heated from room temperature to 1000.0 °C in an air atmosphere.

## 3. Results

### 3.1 Characterization of HNTs@A-171

**Fig. 2:**
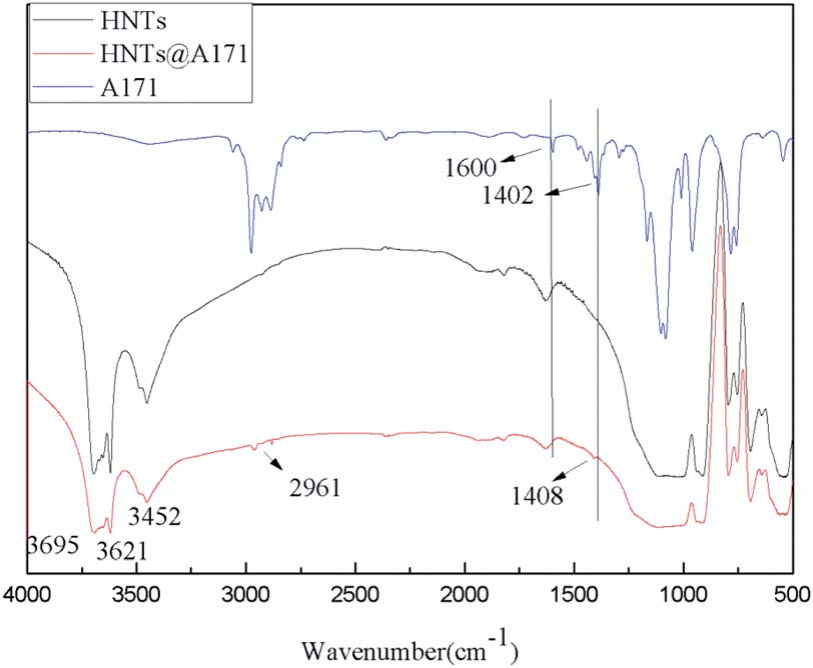
FT-IR spectra of HNTs and HNTs@A-171.

As can be seen from Fig. 2, the HNTS sample has a strong hydroxyl stretching vibration absorption peak near 3695 cm^−1^ and 3621 cm^−1^, which is attributed to the symmetric stretching vibration of the hydroxyl group on the inner surface and the stretching vibration of the internal hydroxyl group located between the silica–oxygen tetrahedron and the alumina–oxygen octahedron respectively. 3430 cm^−1^ and 1647 cm^−1^ are the bending vibration absorption peaks of hydroxyl groups adsorbed by HNTs, and 1300–400 cm^−1^ are the absorption peaks of Si–O and Al–O. In addition, in the infrared spectra of A-171, stretching vibration absorption of C=C and shear vibration of C=C are at 1600 cm^−1^ and 1402 cm^−1^ respectively, while non-plane swing vibration absorption of trans C–H and non-plane swing vibration absorption of CH_2_ in Si–C=C are at 1020–1000 cm^−1^ and 980–950 cm^−1^ respectively.^57^

However, the infrared spectrum of HNTs@A-171 changed, the symmetric stretching vibration peak of methyl appeared at 2961 cm^−1^, and the in-plane bending vibration absorption peak of vinyl appeared at 1408 cm^−1^,^1,56^ however, because the absorption peaks at 1600 cm^−1^ and 1020–1000 cm^−1^ and 980–950 cm^−1^ were repeated with the absorption peaks of HNTS, they could not be observed (Fig. 2).

**Fig. 3:**
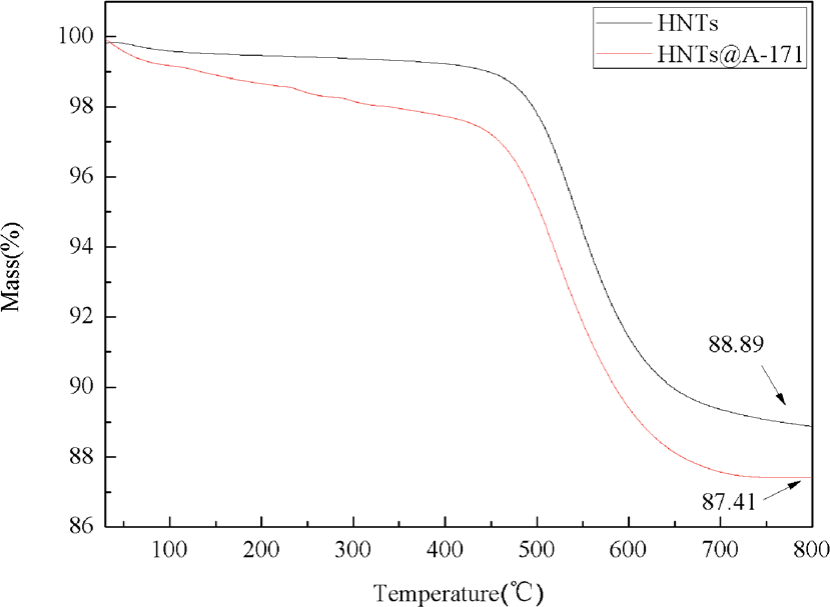
TG curves of HNTs and HNTs@A-171.

Fig. 3 shows the TG curves of HNTs and HNTs@A-171. The initial decomposition temperature (5.0 wt% mass lost) of HNTs is 513.6 °C. And, the initial decomposition temperature of HNTs@A-171 is 503.3 °C, which is lower than that of HNTs. In addition, It can be seen from the Fig. 3 that the thermal stability of HNTs@A-171 becomes worse, this indicates that A-171 has been covalently bound to surface of HNTs. The char yields at 800 °C of HNTs and HNTs@A-171 88.89 wt% and 87.41 wt%, respectively.

### 3.2. Flame retardancy

LOI and UL-94 were used to evaluate the flame retardancy of SiF. The LOI and UL-94 test results of SiF are shown in Table 2. The LOI of SiF without EG was 24.2, and the grade of UL-94 was V-2. Compared with the blank sample, after adding 10 wt% EG or 10 wt% HNTs@A171, the LOI improved by 44.63% and 20.66%, respectively, and UL-94 reached V-0 and V-1, respectively. Thus, EG shows a better LOI than HNTs@A-171. To further improve the flame retardancy of SiF, HNTs@A-171 was used to replace part of the EG, and the flame retardancy of EG/HNTs@A-171 SiF was studied. When 1 wt%, 3 wt%, or 5 wt% HNTs@A-171 was added, the LOI increased from 35.0 to 35.6, and UL-94 remained at V-0. Compared with the addition of EG alone, the amount of EG decreased from 10 wt% to 5 wt% and the LOI increased by 1.7% when HNTs@A-171 was added, indicating that the addition of HNTs@A-171 could improve the flame retardant performance of SiF and had a synergistic flame retardant effect with EG.

**Table 2:** The mechanical properties and flame retardancy of SiF

Sample	LOI/%	UL-94	Tensile strength/kPa	Elongation at break/%
SiF0	24.2 ± 0.2	V-2	60 ± 5	81.16 ± 9
SiF1	29.2 ± 0.3	V-1	100 ± 7	90.43 ± 8
SiF2	35.0 ± 0.3	V-0	120 ± 8	127.59 ± 6
SiF3	35.6 ± 0.2	V-0	140 ± 6	124.74 ± 8
SiF4	35.6 ± 0.2	V-0	140 ± 4	122.6 ± 5
SiF5	35.6 ± 0.3	V-0	130 ± 5	126.55 ± 6

When EG encounters an open fire, it will rapidly expand to form a carbon layer on the surface of SiF and generate a nonflammable gas to prevent the combustion of SiF. HNTs are nanotubes composed of Al_2_O_3_, SiO_2_ and H_2_O, where H_2_O is in the interlayer of the HNTs.^58^ The nanotube structure can enhance the strength of SiF and carbon layer,^55^ thus improving the LOI of SiF. In addition, the endothermic release of crystal water from HNTs also contributes to LOI.

### 3.3. Fire hazard analysis

Cone calorimeter is an instrument designed to measure the heat release from material combustion according to the principle of oxygen content. Key flame-retardant parameters, such as ignition time, heat release rate, total heat release rate, smoke production rate, total smoke production rate, CO release rate, CO_2_ release rate, and mass loss rate, can be directly obtained by cone calorimetry. In addition, the fire growth index (FGI) and fire performance index (FPI) can be obtained from the ignition time, peak heat release rate, and total heat release.^59–61^

#### 3.3.1. Time to ignition (TTI)

TTI is an important parameter for the evaluation and comparison of the fire resistance of materials. The TTI of SiF is shown in Table 3. With the addition of EG and HNTs@A-171 samples, the TTI was improved. When 10 wt% EG or 10 wt% HNTs@A-171 was added, the TTI reached 125 s and 87 s, respectively. Compared with that of the blank sample, the fire resistance of SiF was significantly improved. However, when HNTs were used to replace part of the EG, the TTI decreased significantly compared with that when EG was added alone. When the ratio of EG to HNTS@A-171 was 9 wt% : 1 wt%, the TTI of the sample was only 42 s. With an increase in the amount of HNTs@A-171, the TTI increased. When the ratio of EG to HNTs@A-171 was 1 wt% : 1 wt%, the TTI of the sample reached 56 s. The addition of EG effectively inhibited the ignition time of SiF, which can form an effective carbon layer at the beginning of heating, reduce heat conduction, decelerate the degradation of the SiF main chain, and thus improve the TTI. The addition of HNTs@A-171 limited the expansion ratio of EG when it was heated, resulting in the significant decrease in the TTI of SiF. However, with the increase in HNTs@A-171, HNTs can effectively form a thermal insulation layer to inhibit the degradation of SiF, thus increasing the TTI of SiF.

**Table 3:** Flame retardant characteristic parameters of SiF

Sample	TTI (s)	TPHRR (s)	TSPR (s)	pkHRR (kW m^−2^)	THR (MJ m^−2^)	TSR (m^2^ m^−2^)	SPR (m^2^ s^−1^)	Mass (%)	FGI (kW m^−2^ s^−1^)	FPI (m^2^ s kW^−1^)
SiF0	38	84	74	106.97	58.73	18.90	0.066	56.54	1.27	0.36
SiF1	87	141	120	81.15	44.3	13.60	0.051	63.2	0.57	1.07
SiF2	125	172	161	63.9	39.74	12.26	0.027	65.40	0.37	1.96
SiF3	42	121	101	34.9	21.05	9.12	0.022	64.12	0.17	1.12
SiF4	50	113	110	35.6	16.44	9.82	0.026	65.95	0.15	1.40
SiF5	56	127	110	36.6	21.16	11.49	0.026	67.70	0.17	1.53

#### 3.3.2. Heat release rate (HRR)

The heat release rate per unit area was measured after the sample was ignited with the sample preset under the incident heat flux intensity. The heat release rate is the most important performance parameter for characterizing fire intensity, and its maximum value is the peak heat release rate (pHHR). The magnitude of pHHR represents the maximum degree of heat release from materials during combustion. The greater the HRR and pHHR are, the greater the combustion heat release of the sample, and the greater the fire hazard.^62,63^

**Fig. 4:**
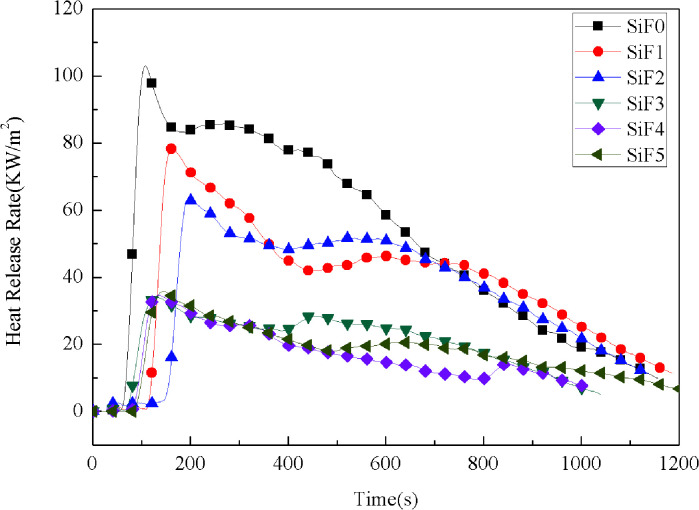
Heat release rate curves of SiF at a flux of 35 kW m^−2^.

Fig. 4 shows the heat release rate of SiF. The heat release rate of SiF decreased significantly after the addition of EG and HNTs@A-171. When 10 wt% EG was added, the peak heat release rate decreased from 106.97 kW m^−2^ to 63.90 kW m^−2^, a decrease of 40.26%. When 10 wt% HNTs@A171 was added, the peak heat release rate decreased from 106.97 kW m^−2^ to 81.15 kW m^−2^, a decrease of 24.13%.When HNTs@A-171 replace part of EG, the heat release rate reached the lowest value of 34.9 kW m^−2^, compared with blank SiF and EG-treated SiF and HNTs@A-171-treated SiF, the heat release rate decrease of 67.42% and 45.38% and 56.99%, respectively. The above results showed that HNTs@A-171 combined with EG could significantly improve the heat release rate of SiF. Both EG and HNTs@A-171 improved the flame retardancy of SiF mainly through the flame retardant mechanism of the condensed phase. In terms of microstructure, HNTs@A-171 are fibrous, and the modified HNTs can form an effective spatial network with the SiF matrix,^64,65^ which limits the expansion ratio of EG, thus solving the popcorn effect of EG, improving the carbon layer strength of EG, and significantly reducing the heat release rate of SiF.

#### 3.3.3. Total heat release (THR)

THR is the sum of the heat released by a material from ignition to flame extinction under the preset incident heat flux intensity. The combination of HRR and THR can be used to better evaluate the flammability and flame retardancy of a material, making the study of fire more objective and comprehensive.

**Fig. 5:**
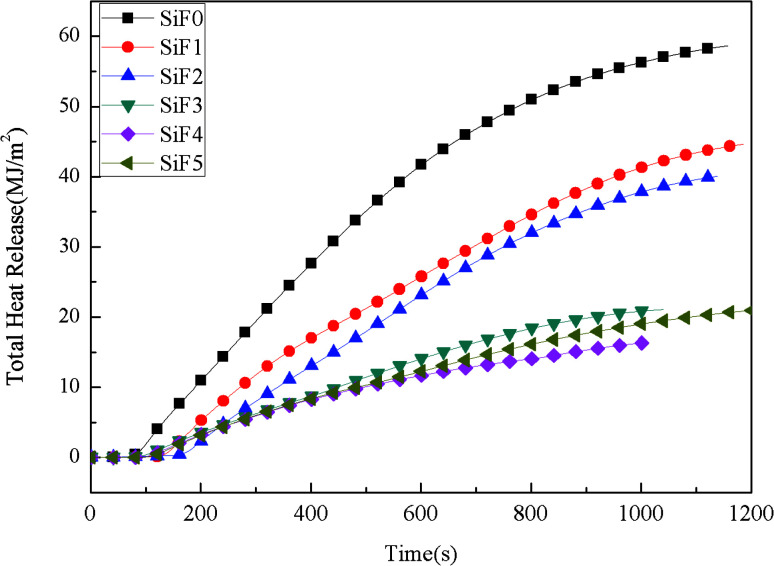
Total heat release curves of SiF at a flux of 35 kW m^−2^.

Fig. 5 shows the total heat release of SiF. The total heat release of SiF without a flame retardant reached 58.73 MJ m^−2^. When 10 wt% EG was added, the total heat release of SiF decreased to 39.74 MJ m^−2^, a decrease of 32.33% compared with that of the blank sample. When 10 wt% HNTs@A-171 was added, the total heat release of SiF decreased to 44.30 MJ m^−2^, a decrease of 24.57% compared with that of the blank sample. After using HNTs@A-171 to replace EG in a certain proportion, the total heat release of SiF decreased to 16.44 MJ m^−2^, and compared with that of the SiF with EG or HNTs@A-171 alone, the total heat release further decreased by 58.63% and 62.88%, respectively. In terms of total heat release, the addition of HNTs@A-171 had a synergistic effect with EG to improve the carbon layer density of EG and reduce the total heat release of SiF.

#### 3.3.4. Mass loss (ML)

**Fig. 6:**
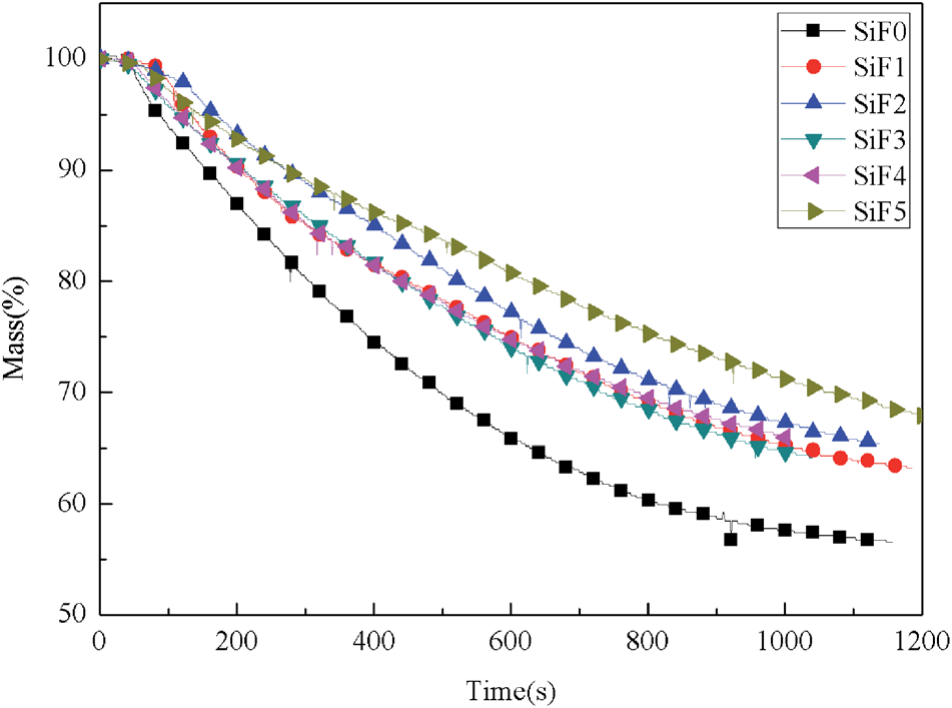
Mass-time dynamic curves of SiF at a flux of 35 kW m^−2^.

The mass loss can be used to determine the residual mass at different times, which is convenient when analyzing the cracking behavior of samples directly. Fig. 6 shows the mass-time dynamic curve of the samples at a heat flow of 35 kW m^−2^. As seen from the figure, the mass loss of blank SiF was faster than that of samples with EG or HNTs@A-171 added. After addition of the flame retardants, the mass residues of the samples were 63.2%, 65.40%, 64.12%, 65.95% and 67.70%, respectively. Compared with that of the blank SiF, the mass residues of the samples increased by 15.67% upon addition of only EG. After a portion of the EG was replaced by 1 wt% HNTs@A-171, the mass residual amount decreased compared with that after the addition of EG alone. However, with an increase in HNTs@A-171, the residual amount of the sample further increased. This indicated that the mass residual rate of SiF could be improved by increasing the amount of HNTs@A-171.

#### 3.3.5. Smoke production rate (SPR)

**Fig. 7:**
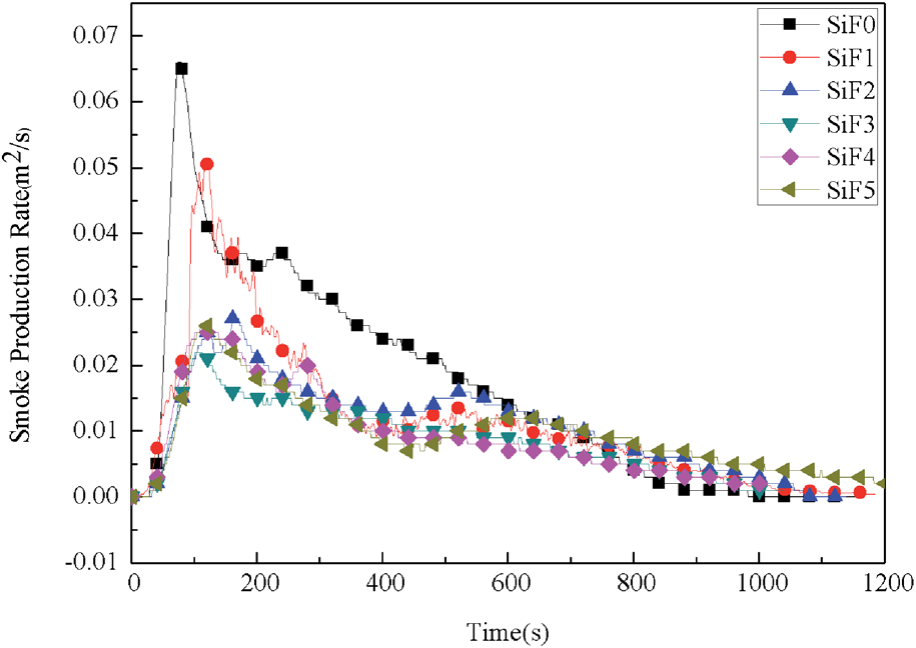
Smoke production rate curves of SiF at a flux of 35 kW m^−2^.

Smoke increases the risk of suffocation, which is more deadly than the heat from a fire. Fig. 7 shows the change in the SiF smoke release rate with time. The pure SiF smoke production rate peaks at 0.066 m^2^ s^−1^, and the SiF smoke production rate reaches 0.027 m^2^ s^−1^ after the addition of EG. Replacing a portion of the EG with HNTs@A-171 can improve the smoke release rate mainly for the following three reasons. First, when EG is heated, it expands rapidly. The formed carbon layer can inhibit the transfer of heat to the silicone rubber foam matrix, prevent the combustion of SiF, and inhibit the transfer of smoke particles to the air during combustion, thus reducing the smoke production rate. Second, HNTs can enhance the strength of the EG expanded carbon layer, inhibit the entrance of oxygen into the blend matrix during combustion, and further prevent the SiF matrix from burning. Third, the crystalline water between the HNTs@A-171 layers is released after heating, which can reduce the subsurface temperature of the SiF. Therefore, the presence of HNTs@A-171 can effectively inhibit flue gas generation during SiF combustion.

#### 3.3.6. Total smoke release (TSR)

**Fig. 8:**
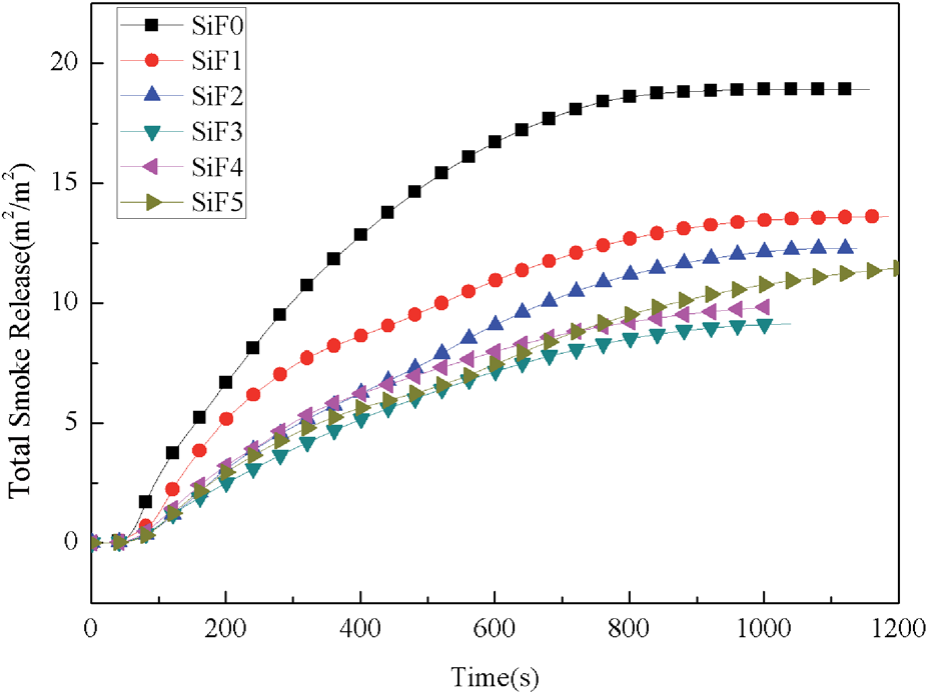
Total smoke release curves of SiF at a flux of 35 kW m^−2^.

Fig. 8 shows the total smoke emission of SiF at a heat flux of 35 kW m^−2^. Compared with that of the blank sample, the total smoke release decreased significantly during the whole combustion process. In addition, compared with that of EG, the total smoke release decreased significantly with the addition of HNTs@A-171. The total smoke releases of SiF0, SiF1, SiF2, SiF3, SiF4 and SiF5 were 18.90 m^2^ m^−2^, 13.60 m^2^ m^−2^, 12.26 m^2^ m^−2^, 9.12 m^2^ m^−2^, 9.82 m^2^ m^−2^ and 11.49 m^2^ m^−2^, respectively. In summary, EG can effectively inhibit the smoke release rate of SiF, which is beneficial for reducing the risk of fire. Added HNTs@A-171 can form an effective collaboration with EG to further reduce the total smoke release.

#### 3.3.7. Fire growth index (FGI) and fire performance index (FPI)

The fire growth index refers to the ratio of the peak heat release rate of a material to the time of the peak heat release, as shown in eqn (1). The value of FGI reflects the ability of the material to respond to heat. With a higher value of FGI, the material will be ignited rapidly once subjected to strong heat, reaching the peak heat release rate, causing rapid spread of the fire and increasing the risk of fire. The fire performance index is the ratio between the material ignition time and the peak heat release rate, as shown in eqn (2). The smaller the FPI is, the more likely the material is to crash after fire, and the greater the fire risk will be.1
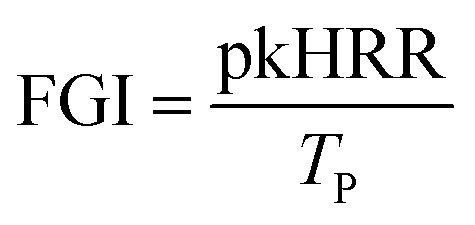
2
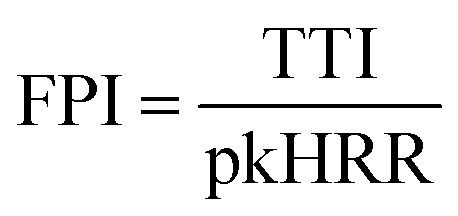


**Fig. 9:**
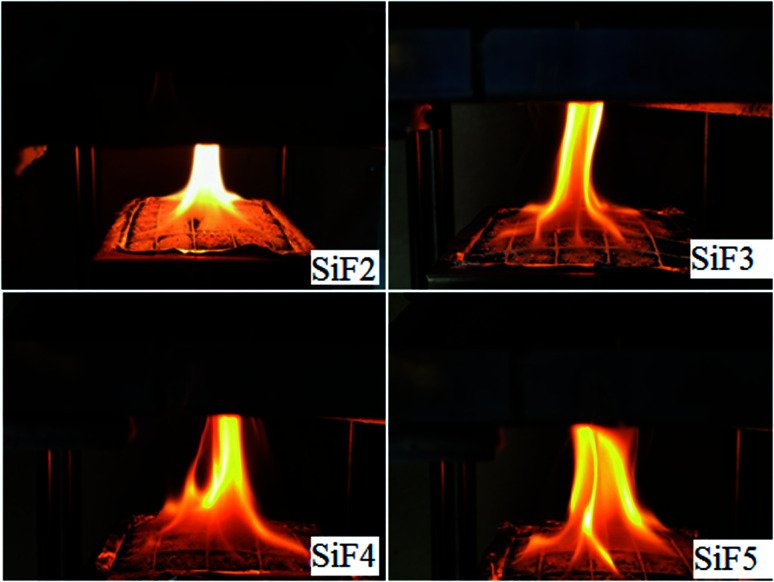
Digital images of the moment of ignition of SiF.

As seen from Table 3, compared with the values obtained after addition of flame retardants, the FGI and FPI of the blank silicone foam sample were the largest and the smallest. When EG or HNTs or EG/HNTs@A-171 was added, the FGI of SiF decreased significantly, while the FPI increased significantly, indicating that both EG and EG/HNTs@A-171 could reduce the fire risk of SiF. However, after the addition of EG/HNTS@A-171, compared with the addition of EG alone, the SiF had a slight increase in fire risk because HNTS@A-171 increased the strength of the EG carbon layer, and a large amount of decomposed combustible gas was covered by the carbon layer and could not be released. However, after the crystallization water between the HNTS@A-171 layers was heated, it uniformly overflowed from the sample surface, resulting in the appearance of uniform pores in the originally completed carbon layer, which slightly increased the fire risk. Immediately after the fire was lit, as shown in Fig. 9, the fire area of SiF3, SiF4 and SiF5 significantly increased at first, but SiF2 had a bright flame, and the burning temperature was high.

#### 3.3.8. CO yield and CO_2_ yield

**Fig. 10:**
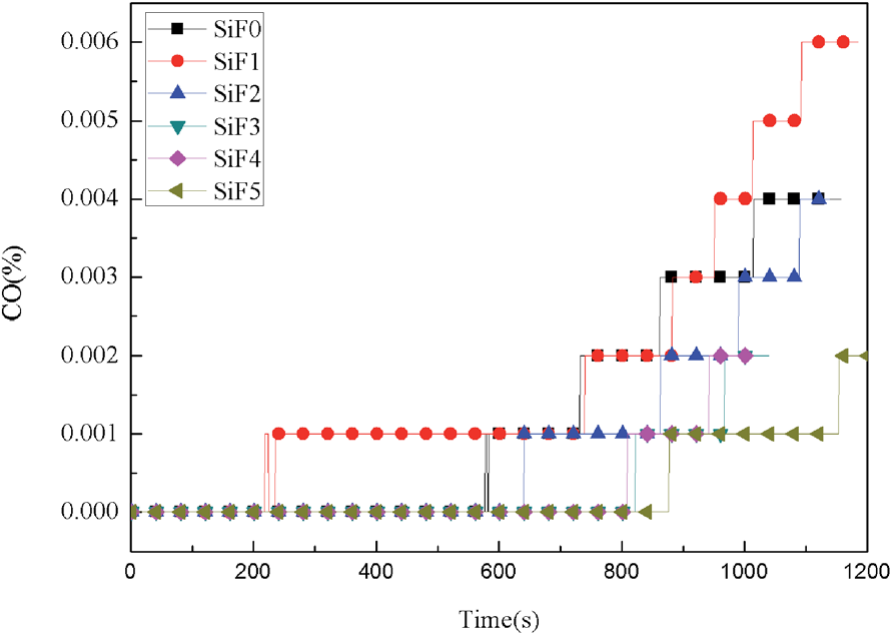
CO yield curves of SiF at a flux of 35 kW m^−2^.

**Fig. 11:**
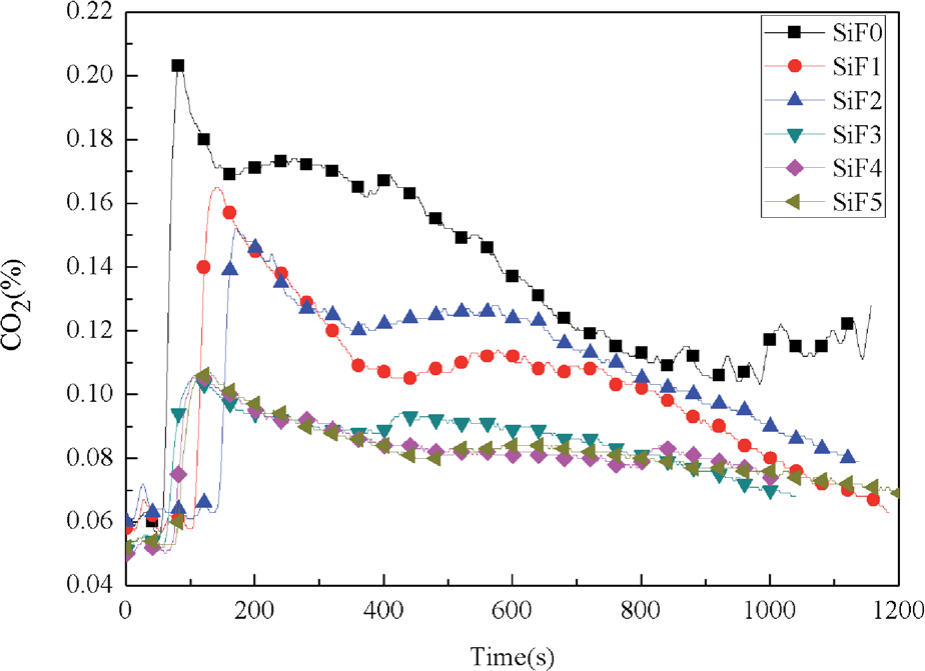
CO_2_ yield curves of SiF at a flux of 35 kW m^−2^.

Many poisonous gases are produced in the process of material combustion. It is very important to control the production of these gases. Fig. 10 and 11 show the CO and CO_2_ yields during the SiF combustion process. As seen from Fig. 10, except for the sample with added HNTs@A-171, before 580 s, SiF did not release CO. Because, as the combustion progressed, the protective layer formed by EG and HNTs@A-171 was strengthened, so oxygen could not penetrate into the residual SiF matrix, and the SiF matrix could not fully burn and thus began to release CO. However, only adding HNTs@A-171 will release CO before 580 s, because HNTs can not form a heat insulation layer similar to EG, resulting in the initial heat, silicone rubber foam decomposition products can overflow. In addition, we also observed that the addition of HNTs@A-171 could reduce the CO production rate from 0.004% to 0.002%.

After the addition of EG and EG/HNTS@A-171, the CO_2_ yield also decreased significantly. The maximum CO_2_ yields of SiF0, SiF1, SiF2, SiF3, SiF4 and SiF5 were 0.203%, 0.165%, 0.152%, 0.105%, 0.106% and 0.107%, respectively. This shows that the combustion state of SiF can be changed when EG and HNTS@A-171 are used together, and HNTs@A-171 addition can enhance the expanded carbon layer of EG, inhibit combustion of the samples, and reduce the generation of CO and CO_2_.

### 3.4. Photographs of char residue

**Fig. 12:**
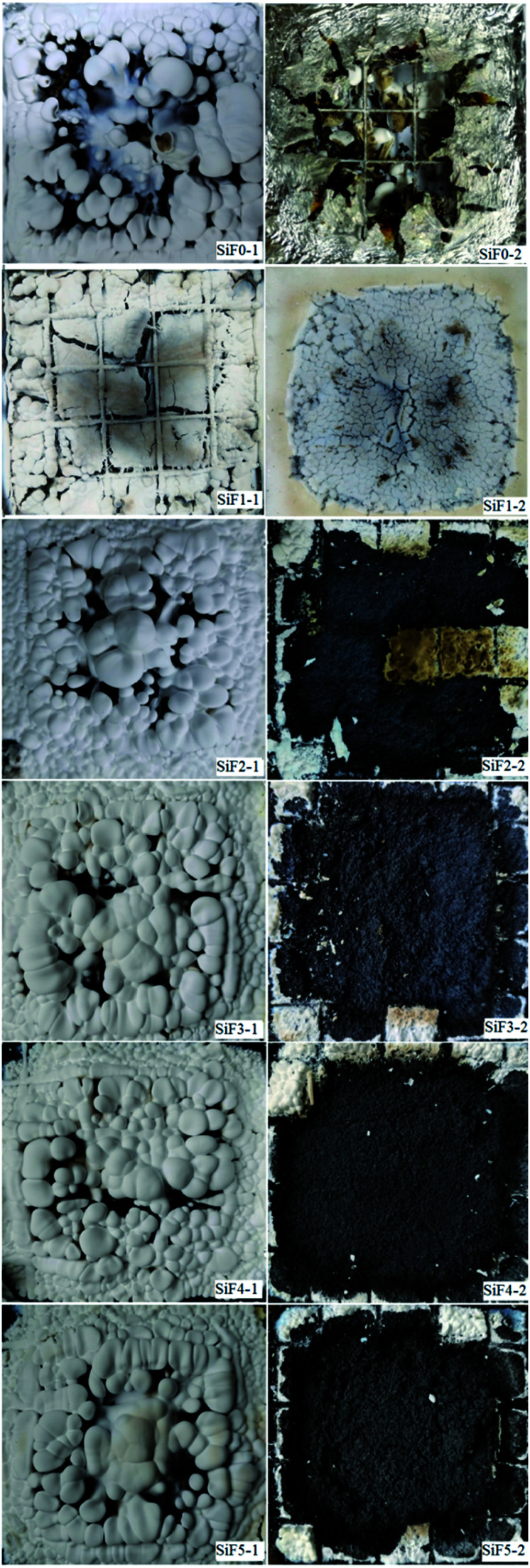
Digital images of flame-retardant SiF samples.

Fig. 12 shows a series of digital images of cone calorimeter residues. As shown in Fig. 12, with the addition of EG and EG/HNTs@A-171, the white residue covering the carbon surface increased significantly. However, when HNTs@A-171 was added only, the white residue on the surface of the silicone rubber foam sample decreased, which was due to the reinforcement of the matrix carbon layer by the element, thus inhibiting the migration of the white residue. When the surface cover was removed, we found that all the blank samples were burned and that the foil protective film was burned through. After adding EG, the carbon residue remained but was relatively loose. After adding HNTs@A-171, the density of the silicone foam samples improved, but with an increase in HNTs@A-171, some cracks appeared in the carbon residue, mainly HNTs@A-171, to increase the strength of the carbon layer. The pyrolysis material and HNTs@A-171 layer between the crystallization water did not overflow smoothly from the EG carbon layer and heating for a long period of time led to the formation of a carbon layer crack. These cracks that form upon the addition of HNTs@A-171, increase the silicone foam peak heat release, total smoking rate and smoke production rate. Further analysis of the residual carbon will be performed in a follow-up study.

The left side is the residual carbon surface of the sample after CCT, and the right side is the internal residual carbon.

### 3.5 Thermogravimetric analysis(TGA)

**Fig. 13:**
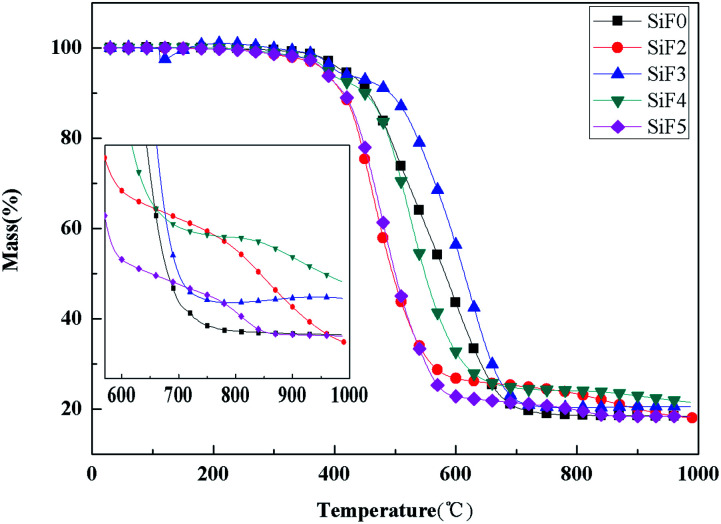
TG curves of flame retardant SiF blents at constant heating rate of 20 K min^−1^.

**Fig. 14:**
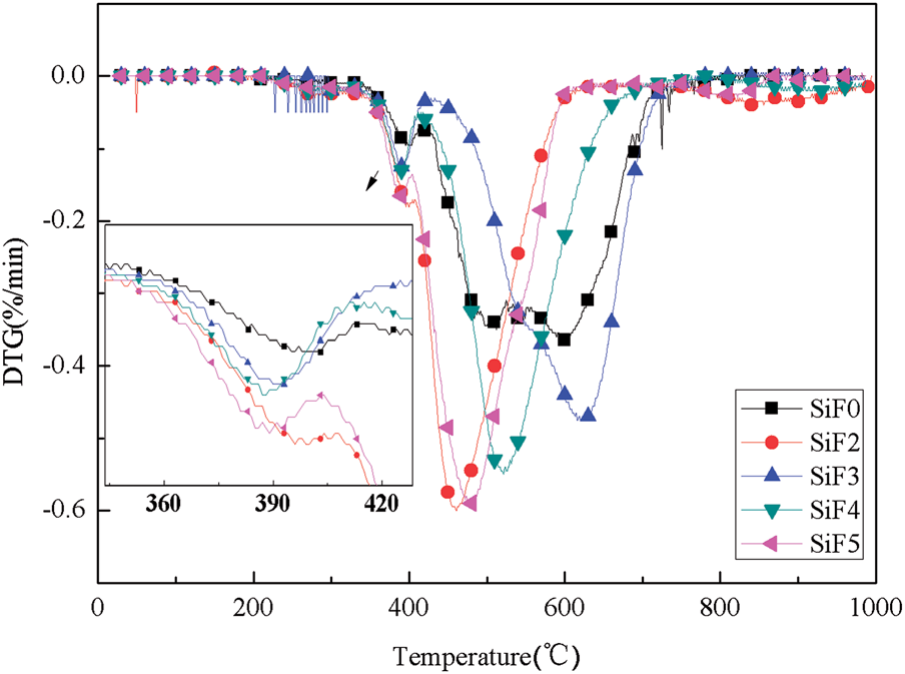
DTG curves of flame retardant SiF blents at constant heating rate of 20 K min^−1^.

Fig. 13 and 14 show the thermogravimetric analysis (TG) and derivative thermogravimetric analysis (DTG) curves for SiF, respectively. The 5% mass loss temperature as initial decomposition temperature and Table 4 shows the main characteristic parameters of TGA. From the initial decomposition temperature, the initial decomposition temperature of the SiF3, SiF4 and SiF5 decreased. The above phenomenon shows that the combined use of EG and HNTs@A-171 can decompose at a lower temperature to form a protective layer to protect the SiF.

**Table 4:** TG parameter of SiF composites

Sample	*T*_5_/°C	*T*_1max_/°C	*T*_2max_/°C	*T*_3max_/°C	PMLR_1_%/min	PMLR_2_%/min	PMLR_3_%/min	Residues at 1000.0 °C/%
SiF0	414	395	497	—	0.095	0.345/0.365	—	18.46
SiF2	354	395	461	856	0.18	0.6	0.04	17.99
SiF3	403	390	620	—	0.125	0.475	—	20.56
SiF4	389	388	521	—	0.135	0.55	—	21.53
SiF5	383	389	478	800	0.17	0.59	0.03	18.37

According to TGA and DTG curves, SiF0, SiF3 and SiF4 have two thermal weightlessness stages, which are 300–450 °C and 450–650 °C respectively. SiF2 and SiF3 have three stages of thermal weightlessness, which are 300–450 °C, 450–650 °C and 800–900 °C, respectively. At 300–450 °C, the Si–C bond in SiF was broken, while at 450–650 °C, the Si–O bond in the main chain was broken. When EG is added alone, the carbon layer formed has a low intensity and is easy to be broken under flame disturbance, resulting in oxidation combustion of the carbon layer at 800–900 °C. When EG and HNTs@A-171 are added at 9 : 1 and 7 : 3, the maximum weightlessness temperature of the second stage reaches 620 °C and 521 °C. Compared with SiF0, the maximum weightlessness temperature of the second stage is increased by 123 °C and 24 °C, respectively. Moreover, no pyrolysis occurs between 800 °C and 900 °C, indicating that HNTs@A-171 can improve the compactness of carbon layer and thus improve the thermal stability of SiF.

From the point of the sample rate of carbon residue, at 1000 °C, adding EG alone, because the carbon layer under high temperature pyrolysis carbon residue decreased, but when adding A certain amount of HNTs, the residual carbon amount to 21.53%, but with the increase of proportion of HNTs@A-171, the residual carbon below SiF0 again, this is because the increase HNTs@A-171, reduced the content of EG, A small amount of EG is insufficient to form A thick layer of carbon, in 800–900 °C when the residual carbon oxidation further decomposition.

### 3.6. Mechanical properties

Mechanical properties directly affect the use of materials. Table 2 shows the tensile strength and elongation at break with or without the addition of EG and HNTs@A-171 and HNTs@A-171. As seen from Table 2, both the tensile strength and elongation at break of SiF increased after the addition of EG and HNTs. When 10 wt% EG was added, the tensile strength of SiF increased by 100% compared with that of pure SiF. When a portion of the EG was replaced with HNTS@A-171, the tensile strength was further increased to 140 kPa, but there was no significant change in the elongation at break. The EG surface contains a large number of hydroxyl groups and carboxyl groups, which can be well dispersed in the SiF matrix to form a continuous-phase carbon layer, thus improving the tensile strength and elongation at break of the SiF. The added HNTs@A-171 can react with SiF to form chemical bonds and a network structure, further increasing the tensile strength of SiF.

## 4. Conclusions

Using silane coupling agent A-171-modified HNTs with EG distribution further improved the flame retardant properties of EG/SiF. The results showed that HNTs@A-171 significantly improved the strength of the EG carbon layer, and the mechanical properties of SiF were also improved. The CCT results showed that the addition of HNTs@A-171 significantly reduced the heat release rate and total heat release amount of SiF. At the same time, the total smoke production and smoke production rate decreased, and the CO and CO_2_ production rates also further decreased. HNTs@A-171 can delay the decomposition temperature of the main chain of the SiF. In conclusion, HNTs@A-171 is an effective method to improve the performance of EG-treated SiF, and HNTs@A-171 and EG have synergistic flame-retardant effects.

## Author contributions

All authors contributed to the innovation. P. Q. T. and D. J. conceived and designed the experiments; P. Q. T. and L. L. performed the experiments; K. F. R. and S. S. Y. performed characterization and analysis of the experiments; P. Q. T., K. F. R. and L. J. wrote the paper. All authors have read and agreed to the published version of the manuscript.

## Conflicts of interest

There are no conflicts to declare.

## Supplementary Material

RA-011-D1RA01409A-s001
