# Supplementary material for: Flame retardancy effects between expandable graphite and halloysite nanotubes in silicone rubber foam
Source: RSC Adv. 2021 Apr 13;11(23):13821–31. doi: 10.1039/d1ra01409a (PMC8697518; doi:10.1039/d1ra01409a)
Supplement: RA-011-D1RA01409A-s001 [file RA-011-D1RA01409A-s001.pdf]

Electronic Supplementary Material (ESI) for RSC Advances.
This journal is © The Royal Society of Chemistry 2021

Supplementary Information

Table 1 parameters of EG flame retardant silicone rubber foam

Sample		TTI（s）	pkHRR（KW/m^2）	THR（MJ/m^2）
SiF-EG0%	24.2	30	69.19	43.25
SiF-EG2.5%	28.2	81	70.48	36.61
SiF-EG5%	30.2	109	70.97	38.82
SiF-EG7.5%	32.6	177	73.89	42.73
SiF-EG10%	35.0	145	63.92	39.74
SiF-EG12.5%	36	202	64.64	30.87
SiF-EG15%	36.3	39	50.75	20.72
SiF-EG17.5%	36.3	47	50.04	23.02
SiF-EG20%	37.4	37	49.46	22.82

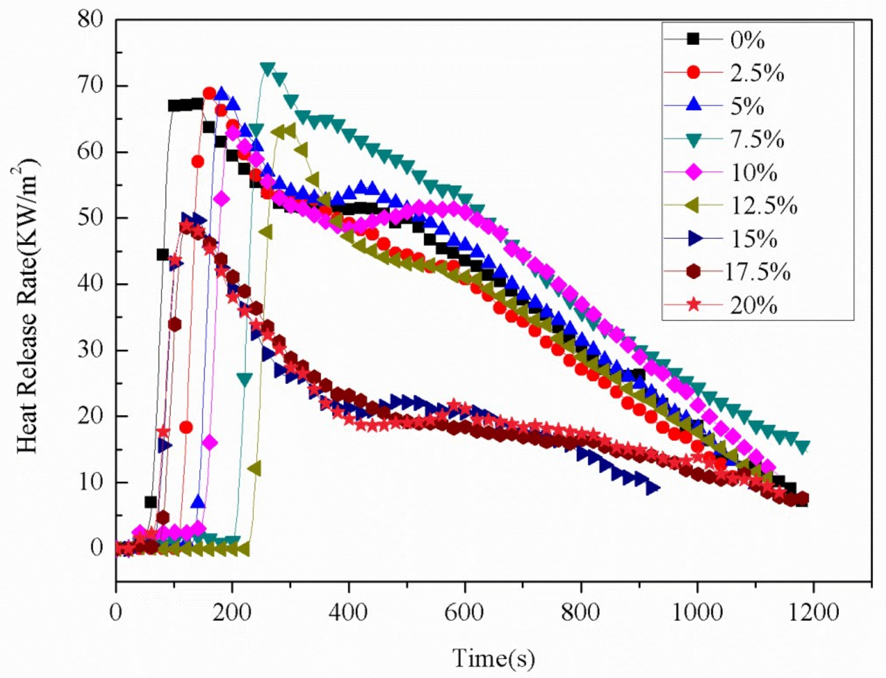

Figure 1. Heat release rate curves of SiF with different amount of EG added at a flux of 35 kW/m^2.

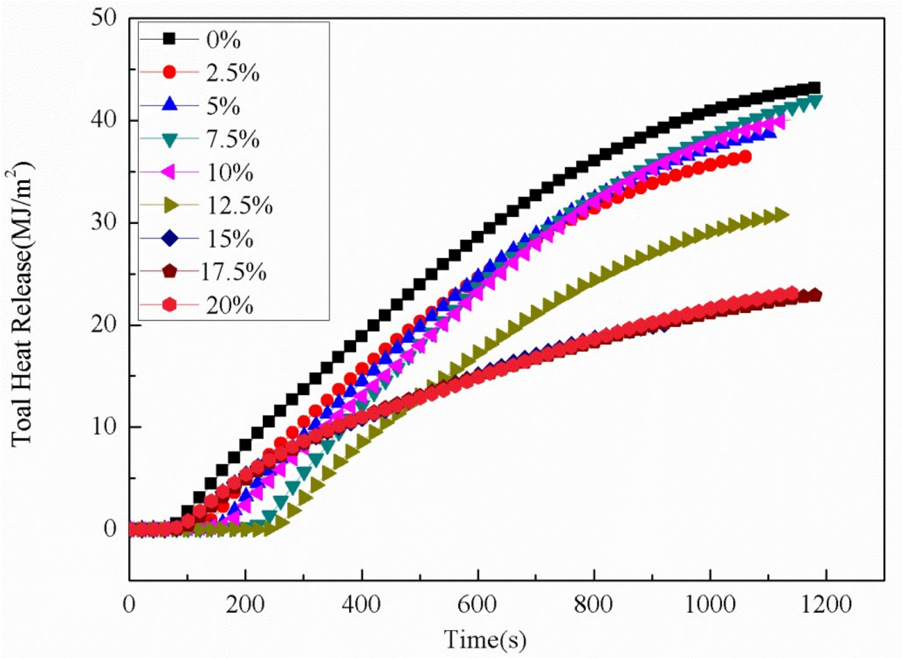

Figure 2. Toal heat release rate curves of SiF with different amount of EG added at a flux of 35 kW/m^2
